# Clinical Experience, Knowledge, Attitudes and Practice of Turkish Pediatric Dentists during the COVID-19 Pandemic

**DOI:** 10.3390/medicina57111140

**Published:** 2021-10-21

**Authors:** Yelda Koç, Serap Akyüz, Damla Akşit-Bıçak

**Affiliations:** 1 Department of Pediatric Dentistry, Faculty of Dentistry, Near East University, Nicosia/TRNC, Mersin 10 99138, Turkey; kocyelda1994@gmail.com; 2 Department of Pediatric Dentistry, Faculty of Dentistry, Marmara University, İstanbul 34854, Turkey; sakyuz@marmara.edu.tr

**Keywords:** anxiety, behavior management, children, coronavirus, COVID-19, infection control, oral health, pediatric dentistry, personal protective equipment, questionnaire

## Abstract

*Background and Objectives*: “Coronavirus Disease 2019” (COVID-19) is a critical public health problem that has affected all fields, including dentistry. The dental management of children has become even more difficult during the COVID-19 pandemic. The purpose of this study was to evaluate the current knowledge, attitudes and practices of Turkish pediatric dentists who have been providing dental treatments to children during the COVID-19 pandemic. *Materials and Methods*: After receiving ethical approval, this cross-sectional study was conducted using the Google Forms online survey instrument. An online questionnaire link was sent to all the members of the Turkish Society of Paediatric Dentistry by e-mail and through social media. Statistical analyses were performed using descriptive statistics and Chi-square test; a *p*-value less than 0.05 was considered statistically significant. *Results:* A total of 200 pediatric dentists participated in this study and most of them (82%) only performed emergency dental treatments, whereas 18.5% performed both emergency and routine dental practices during the COVID-19 pandemic. The vast majority (72.5%) of pediatric dentists prescribed antibiotics and analgesics to their patients who were not cooperative with non-pharmacological behavior management techniques. The findings of the current study showed that the Turkish pediatric dentists had a good level of knowledge about COVID-19, satisfactorily conducted most of the infection control measures before and after the dental treatments, and attached importance to the use of PPEs; however, infection control measures during the dental treatments could be implemented better. *Conclusions:* Along with all precautions, the vaccination of healthcare workers and requesting a recent test result from patients showing a lack of COVID-19 disease before dental appointments could be used as effective infection control measures. Additionally, pediatric dentists should continue to follow local and universal guidelines, and education programmes should be frequently implemented in order to keep their COVID-19 management strategies up to date.

## 1. Introduction

“Coronavirus Disease 2019” (COVID-19) became a critical public health problem after it was first detected in Wuhan, China towards the end of December 2019; the International Committee on Taxonomy of Viruses subsequently reported the name of the new coronavirus responsible for the disease to be SARS-CoV-2. From the beginning of the pandemic, despite all the measures taken to control viral spread, COVID-19 cases emerged worldwide, which indicates that the pandemic has yet to be effectively controlled [1,2]. The SARS-CoV-2 virus is known to be highly contagious, and the possible transmission routes include direct transmission with respiratory droplets through coughing and sneezing, contact with saliva, eye and blood, airborne transmission, indirect transmission via contact with fomites, and fecal–oral transmission [3]. In terms of the risk of COVID-19 exposure, healthcare workers who are constantly in physical contact with patients are among the risk groups. Dentists are recognized to be the highest-risk group among healthcare workers because the required distance for prevention is unobtainable during dental practices, and there is an increased risk of contamination with SARS-CoV-2 through the aerosols and droplets created while performing clinical procedures [1,4,5]. Thus, in order to control viral spread, daily dental practices were reduced/stopped in regions where COVID-19 was widespread, and only emergent/urgent cases were treated [2].

Although COVID-19 vaccinations have been started around the world, widespread immunization requires time and dental professionals are still at high risk of cross infection [5]. Furthermore, pre-symptomatic and asymptomatic patients are both primary sources of SARS-CoV-2 transmission, because at the time of contact, they display no warning signs or symptoms [6]. Pediatric dentists are at higher risk due to the fact that children present nonspecific/mild symptoms, or they could be asymptomatic. Therefore, all child patients and parents should be regarded as potential carriers of COVID- 19 unless confirmed otherwise [7]. Standard infection control procedures applied in daily clinical routines will not be as effective and sufficient for proper protection during the COVID-19 era. The World Health Organization (WHO), the American Dental Association (ADA) and The Centers for Disease Control and Prevention (CDC) recommend that dental practices and hospitals implement rigid and effective infection control protocols to ensure that dentists and dental staff prevent the spread of COVID-19. It is important to prevent the spread of the infectious disease with the precautions applied before the patient comes to the clinic, in the waiting room, during and after the treatment in line with the protocols that dental health personnel must adopt [8]. The WHO has defined this pandemic, of which countries will be at different stages at different times, as having six distinct phases. The planning and treatment options applied in the acute phase of the COVID-19 pandemic may change in the later stages. Therefore, it is difficult to give universal guidelines, which means that it is important to comply with local updated guidelines [7]. At the beginning of the COVID-19 pandemic, the Turkish Association of Paediatric dentistry responded quickly and published guidelines in the Turkish language in order to raise the knowledge and awareness of pediatric dentists in Turkey. Dental teams must follow the instructions and guidelines for better and safer practice during the COVID-19 pandemic [8,9,10].

According to all this information, the purpose of this study was to evaluate the current knowledge, attitudes and practices of Turkish pediatric dentists who have been providing dental treatments during the COVID-19 pandemic, and to compare and interpret the findings with other studies. The hypothesis of this study was that Turkish pediatric dentists have sufficient knowledge, attitudes and practices about COVID-19. It is predicted that the COVID-19 pandemic will ultimately end like the ones before, but the procedures applied/not applied and experiences evaluated will provide valuable learning for future possible epidemics. Additionally, this study aims to share and transfer the experiences, opinions and needs of Turkish pediatric dentists with other pediatric dentists during the pandemic period, and can therefore be considered as a guiding study in this field.

## 2. Materials and Methods

Ethical approval for this cross-sectional study was obtained from the Research Ethics Committee of Near East University (YDU/2020/79-1083). The study was conducted using the Google Forms (Google LLC, Menlo Park, CA, USA) online survey instrument. The sampling frame for this study comprised pediatric dentistry specialists and postgraduate students in pediatric dentistry who worked at a state university hospital, state clinic/center/hospital, private university hospital, or private practice/clinic/hospital in Turkey and the Turkish Republic of Northern Cyprus. The self-administered questionnaire prepared in Turkish was firstly piloted on 20 colleagues in order to ensure its clarity and feasibility. The English language version of the questionnaire can be found in Supplementary File S1 (See Supplementary Materials). After making minor changes, the questionnaire was deemed to be comprehensible and no other corrections were required. First of all, the online questionnaire link was sent to all the members of the Turkish Society of Paediatric Dentistry by e-mail. In addition, pediatric dentists were invited to participate via the Facebook groups of Turkish pediatric dentists as well as WhatsApp. Attempts were made to reach all pediatric dentists working in all cities across the country. Participation in the study was voluntary and identification information was not collected from the participants. Information about the study was included in the form and the participants provided their consent to participate before observing the content of the questionnaire. Thus, participants participated voluntarily in the study. The data collection was performed from June 2020 to September 2020. The questionnaire consists of 40 questions divided into 5 parts in order to assess the knowledge, attitude and practice of Turkish pediatric dentists during the COVID-19 pandemic. The first part of the questionnaire consists of demographic data including the region, gender, age, years of practice, education status and institution of participants. In addition, this part includes questions related to the participants’ working experience during the COVID-19 pandemic. The second part of the questionnaire includes questions aimed at assessing the knowledge of the pediatric dentists related to the COVID-19 pandemic. The third part includes the attitudes of participants when encountering a child patient or parent who had signs or symptoms of acute respiratory tract infection, and attitudes towards personal protective equipment (PPE) usage. The fourth part includes emergency situations encountered by participants during the COVID-19 pandemic. The final part includes infection control measures applied before/during/after dental treatments and participants’ practice measures when treating non-cooperative children during the COVID-19 pandemic.

### Statistical Analysis

In this study, IBM SPSS-21 software was used for statistical analysis. The mean, standard deviation, frequency and percentage distributions of the variables were found with descriptive statistics. With the Chi-square analysis applied to the categorical variables, we observed whether they had a statistically significant difference. As a result of categorical data analysis, those with a *p*-value less than 0.05 were considered statistically significant and are indicated in the tables.

## 3. Results

A total of 200 pediatric dentists participated in this study. The majority of the participants were from Turkey (96%), and the remainder were from the Turkish Republic of Northern Cyprus (4%). Additionally, most of the pediatric dentists were female (86%) and only 14% were male. In total, 47% of all pediatric dentists were between 20 and 30 years of age, 40% were 31–40 years of age, 11.5% were 41–50 years of age and only 3 pediatric dentists were 51 years of age or over. In total, 40% of participants were postgraduate students in pediatric dentistry, whereas 60% were pediatric dentistry specialists. Approximately 57% of all pediatric dentists worked in a state institution and 43.5% in a private institution. Among them, 64% of the study participants were academicians working in either state or private university hospitals (Table 1).

The vast majority of pediatric dentists (96%) reported that their working time decreased and 75% continued to live with their family during the COVID-19 lockdown period. None of the participants had a COVID-19 infection prior to participating in the study. Family members of three of the pediatric dentists had COVID-19 infection. Furthermore, 46 pediatric dentists’ colleagues (healthcare workers in the same institution) also had COVID-19 infections. Only four female pediatric dentists worked in the filiation team and 55% of all participants had attended a webinar on COVID-19 (Table 2). The highest rate of participation in a webinar programmer was found at private university hospitals (77.4%) (*p* < 0.05).

When pediatric dentists were asked about the symptoms of COVID-19, almost all of them knew the main symptoms, such as fever (99.5%), shortness of breath (98%) and dry cough (97%). Approximately 90% of all participants knew about asymptomatic infection joint or muscle pain (82.5%) and sore throat (79%). The least known symptoms were diarrhea (66.5%), sore eyes (51%), vomiting (46.5%), runny nose (37.5%) and skin rash (35%). Only 29.5% of all participants reported all of these as symptoms of COVID-19. Furthermore, pediatric dentists were asked about the transmission routes of COVID-19. All pediatric dentists knew about direct transmission via respiratory droplets through coughing and sneezing. Some participants listed contact with saliva (94%), indirect transmission through contact with fomites (89.5%), airborne transmission (87.5%) and contact with the eye (85%). The least known transmission routes were fecal–oral transmission (58%) and blood (53.5%) (Table 3).

All pediatric dentists stated that information such as “Dental Healthcare workers are at high risk of being infected with COVID-19 when compared with the general population,” and “Aerosol and droplets formed during dental treatment, increase the risk of spread and transmission of COVID-19” were true. Regarding other statements, the vast majority of pediatric dentists said that the information was true, as shown in Table 4. However, 75% of participants said that the statement “Virus detection from saliva samples can be a diagnostic method” was true, and 57.5% of participants acknowledged that the statement “Protection with mask is not recommended for children under 2 years of age and children unable to remove mask without assistance” was true (Table 4).

Common infection control practices used in the participants’ institutions before dental treatment included “establishing pre-control staff at the institution for screening the temperature with non-contact thermometer and checking appropriate use of face masks” (86.4%), “posting signs and posters at the entrance of the waiting room and in the areas visible to patients to provide instructions about social distance, hand hygiene and respiratory hygiene measures” (81.8%) and “in the waiting room, applying social distance rules, and asking some of the patients to wait outside the building if necessary” (81.3%) respectively. Approximately 70% of the participants obtained medical and dental anamnesis from pediatric patients and parents. Other infection control measures were used at rates varying between 70 and 80%. Approximately 57% of participants reported removing toys or reading materials that could be touched by other children in their institutions. All infection control measures used in the institutions of participants before dental treatment are shown in Table 5.

Forty-four pediatric dentists reported that they had encountered a child patient or parent who had signs and symptoms of acute respiratory infection. Among them, 14 (31.8%) pediatric dentists referred these patients to hospital after treating the patient, 28 (63.6%) referred the patients to the hospital with a medical mask without conducting the treatment and 2 (4.5%) refused to treat the patients and asked them to leave the clinic. On the other hand, 156 pediatric dentists reported that they had not encountered a child patient or parent who had signs and symptoms of acute respiratory infection. However, if they were faced with such a situation, 24 (15.4%) pediatric dentists said they would refer these patients to hospital after treating the patient, 110 (70.5%) said they would refer the patient to a hospital with a medical mask without conducting the treatment, and 22 (14.1%) would refuse to treat the patient and would ask them to leave the clinic (Table 6) (*p* < 0.05).

When the pediatric dentists were asked about the dental procedures they practiced during the COVID-19 pandemic, the vast majority (82%) said they only performed emergency dental treatments, whereas 37 (18.5%) performed both emergency and routine dental practices. The most common emergency situations were reported as severe pain caused by pulpal inflammation (94%), abscess or bacterial infection causing localized pain and extraoral swelling (86.5%), luxations, dental avulsions (41%), dental fractures causing pain or soft tissue injuries caused by trauma (35.5%), and the aerosol-free treatment of temporary restoration loss/fractures (27%), respectively. Other emergency situations are also presented in Table 7.

When the pediatric dentists were asked about PPE usage, scrubs (87%), surgical masks (90%), face shields (83%), goggles (71%), disposable surgical gowns (70.5%), disposable gloves (92.5%), and disposable medical caps (83.5%) were found to be used widely in both aerosol-generating procedures (AGPs) and non-AGPs. N95 respirators were used only in AGPs by 21%, and used in both AGPs and non-AGPs by 51%. FFP2 respirators were used in only AGPs by 16%, and were used in both AGPs and non-AGPs by 43.5%. Some PPEs were reported to have never been used by participants, such as P100 respirators (85.5%), elastomeric half masks (79%), FFP3 respirators (64%), waterproof shoe covers (61.5%) and disposable protective coveralls (58.5%). All used PPEs are displayed in Table 8.

One hundred and seventy-four (87%) pediatric dentists thought that their PPE increased the anxiety levels of children. In total, 110 (55%) participants explained their PPE to children by saying that they were wearing an astronaut suit, 52 (26%) said they had become a superhero, 119 (59.5%) explained the reason, and 43 (21.5%) said they did not give any explanation. One hundred and sixty-eight (84%) participants paid attention to the proper order for donning and doffing their PPE, but only 55% of the participants paid attention to removing their own and other dental healthcare workers’ PPE in a separate isolation room. In total, 102/168 pediatric dentists who paid attention to the proper order for donning and doffing their PPE also paid attention to removing their own and other dental healthcare workers’ PPE in a separate isolation room (*p* < 0.05). Approximately 80% of participants encountered burning, stinging, itching, dryness, etc. on their skin due to frequent hand cleaning and the long-term use of PPE. Furthermore, 141/168 (83.9%) pediatric dentists who paid attention to the proper order for donning and doffing their PPE also encountered a situation such as burning, stinging, itching or dryness on their skin due to frequent hand cleaning and the long-term use of PPE (*p* < 0.05) (Table 9).

The infection control practices used by the participants during treatment are displayed in Table 10. In total, 62% of all participants used manual instrumentation, 59.5% continued to make patients’ appointments via phone, WhatsApp and social networks, and 56.5% treated only one patient in a room. Furthermore, 39% of all participants did not let the parent into the treatment room, while 35.5% applied interim therapeutic restorations (ITR), 34.5% used high-volume saliva ejectors, 33.5% applied preprocedural oxidative or antimicrobial mouth rinse, 29.5% used four-handed dentistry, 29% used an aerosol box and 24.5% used slow-speed handpieces. Rarely used methods included dental extraoral suction systems (6.5%), rubber-dam (6%), chemomechanical caries removal systems (4.5%), two before three after hand hygiene (4.5%), and the use of a dental handpiece with anti-retraction function (2.5%). Only 23.5% of the participants reported that they used an air water syringe, and 19% used a high-speed handpiece and ultrasonic instruments (Table 10). When the pediatric dentists were asked about obtaining X-rays from child patients during the COVID-19 pandemic period, 59.5% stated that they obtained only panoramic radiographs, 24% obtained both panoramic and intraoral radiographs, 4% obtained only intraoral radiographs and 12.5% did not obtain any radiographs (Table 10). Approximately 65% of pediatric dentists reported that they had pediatric patients who were not cooperative with non-pharmacological behavior management techniques during the COVID-19 lockdown period (Table 10).

When pediatric dentists were asked about their treatment measures applied to children who were not cooperative with non-pharmacological behavior management techniques during the COVID-19 pandemic period, the vast majority (72.5%) reported that they prescribed antibiotics and analgesics to their pediatric patients. In total, 33% of participants said they conducted atraumatic restorative treatment (ART), 11.5% applied general anesthesia and 8% used physical restraining in order to control children’s sudden movements during dental treatment. It was found that the Hall technique, chemomechanical caries removal, sedation applications, laser applications and silver diamine fluoride (SDF) applications were not widely used (Table 11).

Infection control practices used by the participants after treatment are displayed in Table 12. The infection control measures used were stated as ventilation of the treatment room after each patient (85%), regular disinfection of common areas, door handles, chairs and tables with 0.1% sodium hypochlorite (72.5%), disinfection of reusable PPE with 70% alcohol after each usage (68%), disinfection of commonly used areas such as dental unit, dental light, dental X-ray machine after each patient with 70% ethanol, 0.1% sodium hypochlorite, or 0.5% hydrogen peroxide (66.5%), discharge of medical waste in accordance with the legislation (64.5%), cleaning and sterilization of the dental hand instruments immediately after usage (58.5%) and asking children and parents to use hand sanitizer when leaving the clinic (52.5%). Rarely used measures included fogging system for disinfection (35.5%), ventilation and air-purifying system (16%), ultraviolet radiation system (9%), and high-efficacy particulate air (HEPA) filtration system (7.0%).

## 4. Discussion

This cross-sectional study, which assessed the knowledge, attitudes and practices of pediatric dentists during the COVID-19 lockdown period, is the first to be conducted among Turkish pediatric dentists and one of the first studies of its kind worldwide [11,12,13,14]. Additionally, this study revealed real-life evidence on the efficacy of the guidelines and protocols among Turkish pediatric dentists. In this study, most of the participating pediatric dentists were female (86%) and only 14% were male, which indicates that the number of female pediatric dentists is higher than males in Turkey. Similar results were also reported by other studies [11,12,13,14]. In addition, the vast majority of participating pediatric dentists (87%) were young, between the ages of 20 and 40. This might be because of the web-based nature of the study, and the rate of participation of young people in such studies could be higher because they spend more time on the internet. Moreover, 64% of the study participants were academicians working in either state or private university hospitals; thus, because of their interest in scientific studies, their participation rate might be higher than others.

Along with the closures that occurred in many parts of the world during the COVID-19 lockdown period, dental clinics were also affected, and many of them were closed [15]. The results of this study show that the vast majority of pediatric dentists’ (96%) working time decreased during this period. Similarly, practice closures or reductions have been reported by previous studies [16,17,18,19,20,21,22,23]. Some reports in the literature have demonstrated that most dentists accepted that they were at high risk and were afraid of contracting/spreading the COVID-19 virus to their families [2,18,24,25]. Nevertheless, in this study, 75% of all pediatric dentists continued to live with their families during the COVID-19 lockdown period. Similar findings were presented by Duruk et al. [2] and Hua et al. [20]. The results of this study reveal that, at the time of our survey study, pediatric dentists and people in their surroundings had low rates of COVID-19 disease. Similar observations were also reported in previous studies [17,22,23,24,26]. The reason for this result might be the closure of clinics at the beginning of the pandemic and the disruption to interpersonal contact. 

The present study showed that 55% of all participants attended a webinar on COVID-19. Previous studies [2,11,13,20,27,28,29,30,31,32] showed the various rates of attendance of dentists at an education program on COVID-19. Contrary to these results, some researchers reported insufficient training [12,14,33,34]. In this context, continuous training programs are necessary in order to acquire perfect infection management skills for the present and future possible pandemics.

In order to provide adequate protection, dentists and other healthcare personnel should be aware of the symptoms and transmission routes of COVID-19 disease. Clinical findings of COVID-19 infection in children involve runny nose, fever, shortness of breath, dry cough, muscle or joint pain and gastrointestinal symptoms such as vomiting and diarrhea [35]. Most of the pediatric dentists in this study knew the main symptoms, such as fever, shortness of breath, dry cough, joint or muscle pain, sore throat and diarrhea. However, it was detected that non-respiratory symptoms were not well known. Nearly half of the pediatric dentists knew about sore eyes and vomiting. The least known symptoms were runny nose and skin rash. Cai et al. [36] reported that some COVID-19-positive children did not present respiratory symptoms as the first manifestation. Therefore, pediatric dentists should have information about non-respiratory symptoms as well as the main symptoms of COVID-19. Nevertheless, most of the pediatric dentists in this study were aware of the possible symptoms of COVID-19 that accompany an infection, in accordance with the previous studies [11,12,29,30,31,32,33,37,38,39]. Furthermore, most children can be asymptomatic, and do not show any warning signs to the practitioners; thus, asymptomatic and pre-symptomatic children significantly increase the transmission [6,40,41]. In this study, it was found that the vast majority of pediatric dentists (90%) knew that COVID-19 disease could be asymptomatic.

The potential COVID-19 transmission routes comprise direct transmission with respiratory droplets through coughing and sneezing, contact with saliva, eyes and blood, indirect transmission through contact with fomites, and fecal–oral and airborne transmissions [3]. The results of this study reveal that transmission routes are well-known among Turkish pediatric dentists, but the least known transmission routes were fecal–oral transmission and blood. The aerosols generated during dental treatment, including blood, saliva and organic particles, cause air and environmental pollution in the clinic. Thus, it is very important for dentists to know that the virus can be transmitted through blood. When dental treatments creating aerosols are conducted, the spread of viruses, bacteria and germs increases because of the presence of blood and saliva [32]. In the study of Bekes et al. [12], more than 80% of pediatric dentists specified all the factors related to close contact with the infection. In another study by Moheb et al. [13], most pediatric dentists knew the droplet (92.6%) and direct contact (65.8%) modes, but only 34.2% identified the indirect contact mode of infection. Maru et al. [11] reported that 66.7% of Indian pediatric dentists were informed about the transmission routes of COVID-19 infection. Most of the pediatric dentists in this study were knowledgeable about the possible COVID-19 transmission routes, which is in line with other studies [29,30,31,32,34,37,38,39,42,43] in the literature. On the contrary, Nasser et al. [33] reported poor knowledge among dentists in Lebanon related to transmission routes of the disease. Being conscious about the symptoms and transmission routes of COVID-19 is critical in order to not neglect the necessary measures during dental procedures.

The level of knowledge of participating pediatric dentists was also evaluated by asking about some important information related with COVID-19 besides the symptoms and transmission routes. Among these, all pediatric dentists accepted that dental healthcare workers are at high risk of being infected with COVID-19 when compared with the general population. Most of the dentists in other reports [12,17,26,31,32,33,44,45] expressed a similar opinion. Estrich et al. [26] declared that non-clinical activities in the dental office could be a reason for transmission. Banakar et al. [5] stated that dental settings contribute to the transmission of COVID-19 through AGPs. In the current study, all pediatric dentists stated that the statement “Aerosol and droplets formed during dental treatment, increase the risk of spread and transmission of COVID-19” was true. Similarly, in the study of Candeiro et al. [39], 98.5% of the participants affirmed that COVID-19 can be transmitted during dental procedures. Furthermore, in another study [31], nearly 74% of participants said that the dental treatments performed by using high-speed handpieces and ultrasonic scalers elevate the risk of virus transmission because of the creation of aerosol particles.

In this study, nearly all pediatric dentists knew that children can be asymptomatic or present mild, nonspecific symptoms, and accepted that asymptomatic/pre-symptomatic patients in the incubation period could be carriers of COVID-19. Similar observations were reported by Bekes et al. [12], Arora et al. [31], and Maru et al. [11]. On the contrary, in the study of Nasser et al. [33], 88.3% of Lebanese dentists agreed with the statement “Coronavirus does not infect children”. Ahmed et al. [34] reported that most of the participants (78.7%) in their study knew that COVID-19 affects people from older age groups, but only 7.6% knew that infants could also be affected. In the study of Martina et al. [24], the majority of dentists accepted that adolescents and children had the same infection risk as adults. Asymptomatic carriers are the main cause of transmission and, if present, these mild symptoms can be easily confused with flu-like symptoms [31]. For this reason, dentists should consider each patient as COVID-19-positive and take the necessary precautions during dental treatments, as nearly all pediatric dentists confirmed this statement in our study. Furthermore, pediatric patients present additional risks of transmission due to the use of appliances, the difficulty in using PPE, and patients coming to the clinic with one or more parent [46]. The results of this study show that 98.5% of pediatric dentists accepted that this information was true.

It is important to have knowledge of the fact that wearing any type of mask is not recommended for children younger than 2 years of age and children unable to remove their mask without assistance. This is because of their small airways and the increased risk of suffocation [47]. Unfortunately, only 57.5% of participants knew this information in our study. Moreover, among the detection methods of COVID-19, nucleic acid-based detection has become reliable and rapid, although a negative result does not mean that the possibility of COVID-19 infection can be rejected, and it should not be considered as the only criteria for patient management or treatment determination [48]. In the current study, nearly all pediatric dentists knew that a single negative PCR test result does not exclude the possibility of COVID-19 infection among suspected patients. Together with this, saliva could be a fast, inexpensive and non-invasive method of detecting COVID-19 disease [49]. In this study, 75% of participants agreed that the statement “Virus detection from saliva samples can be a diagnostic method” was true.

Considering the present study, the vast majority of pediatric dentists had a good level of knowledge about COVID-19 disease. Although questions evaluating the knowledge level of dentists might be different among studies, previous studies have also found that dentists have a good knowledge level as well [11,12,28,30,34,50]. The results of this study showed that the knowledge level of dentists about COVID-19 is sufficient, but it is recommended that information should be continuously updated in the following periods according to the course of the disease, in order to optimize the management of COVID-19. 

Many countries have restricted dental treatments and only permitted emergency treatments during the COVID-19 lockdown period; however, these arrangements were not long-sighted or economical. Thus, infection control regimens should be revised for the current pandemic and subsequently for the long-term endemic period [51]. There are important infection control measures and precautions that each institution should implement before conducting dental treatments [51,52,53]. Among these, “posting visual public notices for all visitors at the building entrances including signs and symptoms of COVID-19 and warning not to enter the facility if they are exhibiting any of these symptoms” was implemented by nearly 79% of pediatric dentists’ institutions. In addition, posting signs and posters at the entrance of the waiting room and in the areas visible to patients to provide instructions about social distancing, hand hygiene and respiratory hygiene measures was conducted by 81.8% of participants. Similarly, Moheb et al. [13] reported that nearly 53% of dentists printed and placed patients’ instructions for cough etiquette and social distancing. As another infection control measure, 60% of participants triaged dental patients by phone or online conferencing in order to obtain information on children’s health and COVID-19 risk status, and to determine emergency dental problems in the current study. Furthermore, nearly the same percentage of pediatric dentists completed their patients’ control appointments via phone, WhatsApp and social networks. Similarly, triaging dental patients was reported to have been conducted in previous studies [13,14,16,17,22,23,42,44,54,55]. 

Along with the infection control measures, employing pre-control staff at the institution for screening temperatures with a non-contact thermometer and checking the apropriate use of face masks was conducted by 86.4% of participants in the current study. This finding was found to be consistent with those of previous studies [14,18,21,26,27,54,56], but it contradicted the findings of Cagetti et al. [17], Consolo et al. [22] and Izzetti et al. [16], where taking the temperature of the patients before dental treatments was a practice that was implemented at a lower rate. Moreover, questioning the travel history and presence of symptoms of everyone before entering the building was conducted by 72.2% of participants in this study. Similarly, Ates et al. [54] reported that before the dental treatments, 73.4% asked if the patient had symptoms, shortness of breath, cough or fever. Contrarily, in the study of Duruk et al. [2], only 4% of participants conducted this procedure. In addition, nearly 70% of pediatric dentists obtained medical and dental anamnesis from children and parents. The dental management of children with special needs and medically compromised children requires careful consideration of their health condition as well during the COVID-19 pandemic era [40]. 

As another critical infection control measure, hand washing and/or using alcohol-based disinfectants before entering the operating room should be encouraged for all patients [32]. In our study, placing hand sanitizer and asking children and parents to use it when entering the clinic was conducted by 73.2% of participants. Moheb et al. [13] reported that nearly 93% of dentists offered alcohol hand sanitizers to patients and parents. In another study, asking patients to wash their hands was reported by nearly 78% of dentists in North Italy [17] and nearly 65% of dental hygienists in Italy [42]. According to Al-Khalifa et al. [56], 68% of participants reported patients’ hand washing/sanitizing before going into the waiting area. However, in the study of Putrino et al. [32], the usage of alcohol disinfectant at the entrance for hand cleaning was reported by only 9.7% of participants. Additionally, scheduling the appointments of patients at times not close to each other in order to prevent crowding and the establishment of the time required for disinfection and ventilation was conducted by 71.7% of participants in our study. This finding is in accordance with the studies conducted by Bekes et al. [12], Cagetti et al. [17], Nasser et al. [33], and Consolo et al. [22]. Furthermore, applying social distancing rules in the waiting room, and asking some of the patients to wait outside the building if necessary, was conducted by 81.3% of participants. Similar procedures were also applied in previous studies [13,14,16,17,23,42,56]. 

Ensuring that the pediatric patient comes to the clinic with a single accompanying person was reported by 67.5% of participants in this study. Similarly, Moheb et al. [13] reported that nearly 97% of dentists instructed parents that only one accompanying person was allowed with the child. The results of the study conducted by Bekes et al. [12] showed that nearly 78% of dentists allowed children to come to their appointments with only one accompanying person. According to Allevi et al. [21], one of the most frequently used methods was limiting relatives’ visits (96%). Izzetti et al. [16] also reported that 97.5% of participants discouraged the presence of accompanying people. Lastly, removing toys or reading materials that could be touched by other children was conducted by 56.6% of participants in this study. In line with our findings, dentists in other studies [13,14,16,17,42] removed reading materials and other materials that are not easily disinfected. According to this information, it is obvious that the vast majority of pediatric dentists in this study satisfactorily conducted most of the mentioned infection control measures before the dental treatments. However, procedures including removing all unnecessary materials in the dental office, triaging patients and only allowing one person to accompany the child could be better implemented. 

CDC and ADA guidance reports that active COVID-19 patients and patients exposed to a person with confirmed/suspected COVID-19 infection, and patients who had been in countries under a travel ban, should not be treated in dental clinics. If emergency/urgent dental care is needed, patients should be assessed for COVID-19 symptoms. Patients who have/do not have fever together with signs and symptoms of acute respiratory infection need to go to the emergency department of a hospital for infection control and treatment measures. If patients do not have fever and signs and symptoms of acute respiratory infection, or only have fever, they can be treated at the dental clinic because the fever might because of a dental infection [6,52]. In the current study, among the pediatric dentists who encountered a child patient or parent who had signs and symptoms of acute respiratory infection, nearly 64% of them referred the patient to a hospital with a medical mask without conducting the treatment in accordance with the ADA and CDC guidelines, and 4.5% refused to treat the patient and asked them to leave the clinic. Among pediatric dentists who did not encounter a child patient or parent who had signs and symptoms of acute respiratory infection, nearly 70% reported that they referred the patient to a hospital with a medical mask without conducting the treatment, in line with the recommendations, and nearly 14% refused to treat the patient and asked them to leave the clinic. Moheb et al. [13] reported that nearly 55% of dentists prescribed medication if they faced a patient with fever and no other signs/symptoms of COVID-19 infection. Moreover, researchers also reported that 40% of dentists also chose to control the situation with medicines if they were faced with a patient who did not report any symptoms but the dentist observed signs of respiratory illness. Similarly, Nasser et al. [33] showed that more than 80% of the dentists were afraid to treat a patient suspected of or confirmed as having COVID-19. In another study by Khader et al. [38], nearly 44% of dentists declared that they would refer the patient to the hospital without conducting treatment, 4.6% declared that they would refuse to treat the patient and ask them to leave the clinic, and nearly half of the dentists would treat the patient and ask them to go to the hospital when they were faced with a patient coughing and sneezing in their dental clinic. Furthermore, in the study of De Stefani et al. [29], nearly 66% of dentists said they would have refused to treat a patient suffering from a runny nose and cough.

In our study, among the pediatric dentists who encountered a child patient or parent who had signs and symptoms of acute respiratory infection, nearly 32% of them referred these patients to a hospital after treating the patient. Among dentists who did not encounter a child patient or parent who had signs and symptoms of acute respiratory infection, nearly 15% reported that they referred these patients to a hospital after treating them. Similarly, in the study of Bekes et al. [12], 31% of pediatric dentists had self-confidence in the treatment of suspected COVID infections. Hua et al. [20] reported that nearly 64% of participants wanted to treat patients with suspected/confirmed COVID-19 infection. Maru et al. [11] reported that 57.5% of pediatric dentists had self-confidence in conducting treatment on children with suspected COVID-19; however, 10.80% of pediatric dentists exhibited no confidence. The results of the study conducted by Arora et al. [31] showed that nearly 42% of dentists displayed a positive attitude towards providing emergency dental treatment to COVID19-positive patients. In the study of Becker et al. [44], it was recommended that COVID-19-positive patients’ dental treatments should be performed at dental university hospitals in isolated rooms. Thus, it was strongly recommended that emergent COVID-19 positive/suspected patients should be treated in the hospital’s separate airborne infection isolation rooms (AIIR), which are kept under negative pressure with an HEPA filter. In addition, high-level protection with full usage of PPE is needed. Furthermore, healthcare workers should follow donning and doffing procedures. It is important to know that COVID-19-positive/suspected patients should not be treated in a room that recirculates air within the hospital and without appropriate PPE usage [27,57]. Most of the pediatric dentists in this study referred or considered referring patients who had signs and symptoms of acute respiratory infection to a hospital with medical masks without conducting the treatment, or refused to treat the patient. If the necessary facilities such as AIIR including an HEPA filter are not available, this approach is more acceptable than conducting dental treatments of COVID-19-positive/suspected patients in the same environment with other patients. 

When pediatric dentists were asked about the dental procedures they performed during the COVID-19 lockdown period, the vast majority (82%) said they only performed emergency dental treatments according to Turkish government recommendations. In line with the current report, the majority of previous studies [2,12,13,15,16,22,30,32,39,54,55,58,59] showed that dental practices were reduced to urgent/emergency procedures during the COVID-19 quarantine period. However, Nibali et al. [60] reported that most of the practitioners had completely ended all dental activities. Emergent dental situations include facial swellings, severe dental/facial pain that cannot be controlled with advised medications, traumatic dental injuries such as severe luxation injuries, complicated crown fractures, avulsions of permanent incisors, soft tissue infections, uncontrollable post-operative bleeding, conditions worsening systemic medical conditions, and suspected oral cancer [61]. Furthermore, in our study, the most common emergency situations were reported as severe pain caused by pulpal inflammation (94%), abscesses or bacterial infection causing localized pain and extraoral swelling (86.5%), luxations/dental avulsions (41%), dental fractures causing pain, or soft tissue injuries caused by trauma (35.5%). Ilyas et al. [61] reported irreversible pulpitis, Moheb et al. [13] reported severe pain, infection and trauma, Üstün et al. [62] reported severe dental pain because of pulpal inflammation and swelling, Faccini et al. [58] reported toothache (71.4%), broken restorations (40.4%) and dental trauma (37.3%), Alsaleh et al. [14] reported abscesses (51.8%) and cellulitis (44.6%) in Jordan and abscesses (44.6%) and pulpitis (35.5%) in India, Izzetti et al. [16] reported pulpitis, prosthesis de-cementation and abscesses, and Sinjari et al. [55] reported severe dental pain, swelling and dental trauma as the most common emergency situations managed. Thus, the findings of this study were found to be in accordance with those others [13,14,16,55,58,61,62]. However, differently from our findings, Limbu et al. [63] reported exfoliating mobility (23.3%), acute pulpitis (5.8%) and dental abscesses (22.1%), and Martinho and Griffin [18] reported trauma (82.1%) followed by swelling (81.9%) and pain (76.1%) as dental emergencies.

In order to achieve efficient infection control, dentists and all healthcare workers in the dental clinic should pay attention to appropriate PPE usage while working in the dental clinic, especially during the COVID-19 era [33]. PPE includes disposable scrubs, shoe covers, hair covers, goggles, gloves, gown, masks and face shields. While conducting non-AGPs, work clothes, usage of goggles/face shields, fluid-resistant surgical masks, disposable gloves, disposable aprons and hair covers are recommended. In addition to all these protections, the usage of particulate respirators, disposable gowns, medical protective clothing and shoe covers are required during AGPs. The most effective/protective masks are N99/N100/FFP2/FFP3/N95 masks [64,65]. Scrubs (87%) and surgical masks (90%) were found to be widely used in both AGPs and non-AGPs in the current study. Similarly, Duruk et al. [2], Estrich et al. [26], and Izzetti et al. [16] also reported nearly 86%, 99% and 98% mask usage, respectively. Disposable gloves (92.5%) were found to be used widely in both AGPs and non-AGPs in line with previous studies [2,14,17]. Face shield (83%) usage was found to be similar to that reported by Sinjari et al. [55]. In line with our findings, in studies by Alsaleh et al. [14] and Duruk et al. [2], face shield usage was shown to have high percentages. Furthermore, goggles (71%), disposable surgical gowns (70.5%), and disposable medical caps (83.5%) were found to be used widely in both AGP and non-AGPs in this study. Alsaleh et al. [14] reported a similar rate of goggle usage in India (75.2%). Duruk et al. [2], Izzetti et al. [16] and Sinjari et al. [55] reported that nearly 46%, 80% and 82% of participating dentists used disposable aprons/gowns. Martinho and Griffin [18] showed that 36.8% of participants used protective suits; however, 24% of pediatric dentists used protective coveralls in this study. Moreover, disposable medical caps (83.5%) were found to be widely used in both AGPs and non-AGPs. Similarly, Izzetti et al. [16], and Duruk et al. [2] reported medical cap usage rates of 84.4% and 56%, respectively. 

When we consider the usage of particulate respirators, Alsaleh et al. [14] reported N95 respirator usage rates of 80.4% in Jordan and 87.6% in India. Ammar et al. [37] reported N95/FFP2 usage of 91.7%. Martinho and Griffin [18] reported that the usage rate of N95 respirators was 83.1%. N95 respirator usage was found to be lower in our study, where 21% of pediatric dentists only used AGPs and 51% used both AGPs and non-AGPs. However, lower percentages than in this study were also reported by Duruk et al. [2] (12.36%) and Ahmed et al. [34] (11.6%). In addition, FFP2 respirators were used only in AGPs by 16%, and were used in both AGPs and non-AGPs by 43.5%. Cagetti et al. [17] and Izzetti et al. [16] reported rates of usage of FFP2/FFP3 masks of 54.84% and 15.4%, respectively. Sinjari et al. [55] reported FFP2 usage of nearly 62% and Bekes et al. [12] reported rates of nearly 45% for FFP2 and 60% for FFP3e. Estrich et al. [26] also showed that dental practitioners wore surgical masks over particulate respirators, and replaced the surgical masks more often. Considering PPE usage, it was detected in our study that pediatric dentists attach importance to the use of PPEs. Scrubs, surgical masks, disposable gloves, goggles, face shields, disposable surgical gowns, and disposable medical caps were found to be widely used in both AGPs and non-AGPs. However, the usage of particulate respirators such as N95, FFP2/FFP3 and overshoes should be increased. The reasons for this result could be that the dentists thought that a surgical mask combined with a face shield could offer sufficient protection, the fact that wearing a respirator for long time is not easy, and the high costs of respirators [13]. Furthermore, difficulty in accessing PPE supplies could be another reason [16]. 

Knowing the correct sequence for donning and doffing PPE is also important, besides using the required PPE, among dental practitioners in order to reduce contamination [57,66]. Furthermore, after the dental treatment, patients and the dental team should leave the clinic from separate exits, and all clinical personnel should pay attention to doffing their PPE in the buffer zone [67]. In our study, most of the pediatric dentists (84%) reported that they paid attention to the proper order of donning and doffing their PPEs; however, only 55% paid attention to removing their and other dental healthcare workers’ PPEs in a separate isolation room. Bekes et al. [12] reported that nearly 52% of the dentists knew the correct sequence of donning PPE (gown, mask, eye protection, gloves). Maru et al. [11] showed that 66.5% and 64.8% of Indian pediatric dentists knew the correct sequence of donning (gown, mask, eye protector, gloves) and doffing PPE (gloves, eye protector, gown, mask), respectively. 

The parents should prepare their children mentally prior to dental appointments because the dental team behind the PPE may frighten the child [68]. Successful behavior management is crucial for pediatric dentists because uncooperative, crying children spread more aerosols than calm ones. Thus, in order to reduce the anxiety of children related to the dental team’s PPEs, including face mask, it is recommended that they put them on in front of the children and explain the necessity of this equipment [40]. In our study, most of the pediatric dentists thought that their PPE increased the anxiety levels of children. Furthermore, more than half of participants explained their PPE to their pediatric patients as them wearing an astronaut suit, 26% said they had become a superhero, 59.5% explained the reason of usage and 21.5% did not give any explanation. In another study, nearly 28% of dentists reported that children did not react differently, nearly 32% reported children were interested, nearly 30% reported children were distressed and worried, but nearly 10% of dentists reported that they did not use advanced PPE while treating children [13]. Additionally, extra PPE covers facial expressions and complicates behavior management in children. Thus, PPE could be modified in order to make it more child friendly [14]. Before dental treatment, meeting with the pediatric patient via video call without wearing PPE and introducing PPE with the tell–show–do technique, using colorful/painted gowns, and placing stickers or the dentist’s picture on the PPE were suggested modifications for pediatric dental clinic practices [14].

When we evaluated the infection control measures used during dental treatments, using hand instrumentation, avoiding high-speed handpiece/ultrasonic instruments/air water syringe usage, and the treatment of only one patient in a room were conducted by most of the pediatric dentists. Moheb et al. [13] reported that nearly 85% of dentists used hand instruments as much as possible to avoid aerosols, and nearly 84% minimized the use of a three in one syringe. Additionally, in another study, most of the dentists (88.5%) made the effort to reduce aerosol formation [16]. Ates et al. [54] reported that half of the participants used slow-speed handpieces without water to minimize contamination risk. However, in this study, only 49 pediatric dentists used this approach. The use of a dental handpiece with an anti-retraction function was reported as a preventive measure [42], but only five pediatric dentists reported using those instruments. Among the infection control measures, only 39% of pediatric dentist said they did not allow parents into the treatment room. The reason for this result might be due to the fact young children in particular do not want to be separated from their families during dental treatments. If the child does not want to enter the treatment room alone, a maximum of one parent should be accepted, and other accompanying people should be prevented from entering the treatment room. In addition, chemomechanical caries removal was only conducted by nine pediatric dentists in our study. This might be related to the preference for conventional treatments among pediatric dentists before the COVID-19 pandemic period and the lack of essential dental materials. Furthermore, ITR were conducted by nearly 34% of pediatric dentists, and this result might be because of the delay of routine dental practices and the treatment of emergency dental treatments, mainly abscesses or bacterial infection causing localized pain and extraoral swelling (86.5%). However, since it is not possible to carry out only emergency treatments, approaches that prevent aerosol formation, such as chemomechanical caries removal, ART, ITR and SDF, should be preferred for routine dental treatments after the quarantine period in order to reduce aerosol formation and to prevent contamination with SARS-COV2 during long-term pandemic conditions. Rubber dam usage also reduces the risk of aerosols during the COVID-19 pandemic period. However, in our study, rubber dam usage was found to be unsatisfactory and lower than in other studies conducted by Moheb et al. [13], Izzetti et al. [16], and Sinjari et al. [55]. The findings of Duruk et al. [2] (13.84%) and Ataş and Yıldırım [69] (15.9%) also revealed that rubber dam usage was infrequent. Moheb et al. [13] declared that it is not always feasible to excavate caries only using hand instruments; the application of a rubber dam is not possible for some children, and an air water syringe might be required. Even in such cases, dentists have to do their best to reduce aerosol formation.

Among the infection control methods used during dental treatments, the four-handed technique was used by nearly 30% of participants in this study. Similarly, 37.8% of dentists used this technique in the study of Izzetti et al. [16]. Preprocedural oxidative or antimicrobial mouth-rinse usage was reported by nearly 34% of pediatric dentists in this study. Moheb et al. [13] reported that nearly 56% and Al-Khalifa et al. [56] reported that 47% of dentists preferred patients using antiseptic mouth rinse before dental treatments. Furthermore, high-volume saliva ejector usage was reported by nearly 35% of participants; however, Moheb et al. [13], Duruk et al. [2], and Ataş and Yıldırım [69] reported strong saliva absorber usage rates of 87.2%, 63.79% and 42.4%, respectively. In the study of Martinho and Griffin [18], nearly 17% of participants reported usage of an oral aerosol vacuum. In another study by Estrich et al. [26], 17% of participants said they used an extraoral suction device. Al- Khalifa et al. [56] reported that 59% of participating dentists did not use extraoral suction systems. Unfortunately, in the current study, only 6.5% of participants used a dental extraoral suction system. Lastly, in order to prevent the spread of infections, ensuring good hand hygiene is one of the most efficient methods. A two before (before conducting examination and before performing dental treatment) and three after (after touching the patient, after touching the undisinfected surroundings/equipment, and after touching blood/wound/oral mucosa/damaged skin) hand hygiene system should be conducted by the dental team to strengthen hand hygiene [10]. The results of this study show that nearly 80% of the pediatric dentists encountered a situation such as burning, stinging, itching or dryness on the skin due to frequent hand cleaning and the long-term use of PPEs. However, it was detected that the recommended two before three after hand hygiene protocol was not well known among pediatric dentists in our study. According to our findings, it is obvious that pediatric dentists wash their hands before and after the procedures, and struggle to achieve sufficient disinfection; however, increased hand hygiene following the two before three after hand hygiene protocol should definitely be performed to increase protection against the COVID-19 disease. Therefore, dentists should be familiar with the concept of two before three after hand hygiene. To conclude, in our study, the most crucial control measures, such as the treatment of one patient in a room, not using high-speed handpieces and ultrasonic instruments, not using air water syringes, and preference for hand instrumentation, were found to be more widely used by pediatric dentists than other precautions. However, the lack of implementation of rubber dams, four-handed dentistry, dental extraoral suction systems, high-volume saliva ejectors and two before three after hand hygiene should be increased via education programs. 

Another important point that needs to be considered is the radiographic evaluation of patients during the COVID-19 pandemic period. If radiographs are required from patients, extraoral radiographs should be preferred instead of intraoral radiographs in order not to stimulate the secretion of saliva and coughing [70]. In line with the recommendations, most of the pediatric dentists obtained panoramic radiographs in this study. Similarly, Moheb et al. [13] also reported that nearly 62% of dentist shifted from intraoral to extraoral radiographs, if available. In another study, Ammar et al. [37] reported that nearly 60% of dental academics used extraoral radiographs rather than intraoral radiographs.

The dental management of uncooperative patients during the COVID-19 lockdown period is also an issue that needs to be carefully evaluated. Similar to our study, Moheb et al. [13] reported that the majority of dentists (73%) did not use sedation or general anesthesia during the pandemic period. Additionally, they reported that nearly 70% of pediatric dentists refused to use physical restraining. A similar finding was observed in our study, as only 8% of pediatric dentists used physical restraining in order to control children’s sudden movements. Alsaleh et al. [14] reported that most dentists (71.1% in India and 80.4% in Jordan) did not use N2O sedation for their pediatric patients. For less aerosol production, in India ART (52.1%) and SDF (28.9%), and in Jordan the Hall technique (26.8%) and ART (25.0%), were performed. In the current study, ART (33%) was the most preferred treatment in accordance with Alsaleh et al. [14]; however, SDF and the Hall technique were used less than in the study by Alsaleh et al. [14]. As previously mentioned, minimally invasive treatment approaches were not widely used since most of the pediatric dentists in our study only performed emergency treatments during the lockdown period. However, lockdown periods are not permanent and they are rotative; thus, in order to reduce the risk of transmission, treatments that reduce aerosol formation should be used widely in dental practice.

Among the infection control measures performed after dental treatments, the disinfection of commonly used areas, such as dental units, dental lights and dental X-ray machines, after each patient was conducted by 66.5% of pediatric dentists in this study. Similarly, in the studies of Bontà et al. [42] and Cagetti et al. [17], 74.13% and 80.49% of participants reported that they removed and disinfected disposable protective devices, respectively. In another study, nearly 68% of healthcare professionals said they applied universal precautions for infection control [34]. Khader et al. [38] reported that nearly 94% of participants routinely cleaned and disinfected surfaces that had been in contact with known or suspected patients. On the other hand, the regular disinfection of common areas, door handles, chairs and tables was also deemed to be important and conducted by 72.5% of pediatric dentists in this study. Similarly, in the study of Estrich et al. [26], almost all practicing dentists reported that they disinfected commonly touched equipment and surfaces. Similar observations were reported in previous studies [17,32,33,42,71]. Furthermore, the ventilation of the treatment room after each patient was conducted by 85% of pediatric dentists in our study. In accordance with our findings, in the studies of Bontà et al. [42] and Izzetti et al. [16], nearly 71% and 98% of participants provided room ventilation after dental treatments, respectively. However, Putrino et al. [32] reported that less (14.9%) participants ventilated the treatment room between patients. Air-purifying systems were found to be used by only 16% of pediatric dentists in this study. Martinho and Griffin [18] indicated that 42.3% of endodontists could implement an air-purifying unit. In another study by Huntley et al. [27], 42.5% of the participants reported that they had no access to an air-purifying unit. Among the infection control measures mentioned, the least conducted measures were a fogging system for disinfection (35.5%) and a HEPA filtration system (7%). Similarly, Alsaleh et al. [14] reported that the least conducted measure was the use of HEPA filters in India (41.3%) and in Jordan (8.9%).

Infection control measures after dental treatments are mandatory, and in our study, those precautions were found to be satisfactory applied, such as ventilation of the treatment room after each patient, regular disinfection of common areas in the dental clinic and treatment room, disinfection of reusable PPEs, discharge of medical waste, cleaning and sterilization of the dental hand instruments immediately after usage, and making children and parents use hand sanitizer when leaving the clinic. However, the rarely used measures were fogging systems for disinfection, ventilation and air conditioning systems, ultraviolet radiation systems, and HEPA filtration systems.

## 5. Conclusions

The findings of the current study show that Turkish pediatric dentists had a good level of knowledge about COVID-19, sufficient to perform dental treatments safely and not neglect the necessary precautions during dental procedures. Turkish pediatric dentists satisfactorily conducted most of the infection control measures before and after the dental treatments, and they attached importance to the use of PPEs; however, infection control measures during the dental treatments could be better implemented. At the beginning of the pandemic, most pediatric dentists only performed emergency treatments; however, lockdown periods are not long-lasting and they are rotative. Thus, in order to reduce the risk of transmission, minimally invasive approaches should be used widely in routine dental practices. Along with all precautions, the vaccination of healthcare workers and requesting a recent test result from patients that shows the absence of COVID-19 before dental appointments can be added to the effective infection control measures. Additionally, pediatric dentists should continue to follow local and universal guidelines, and education programs should be implemented frequently in order to keep COVID-19 management strategies up to date. 

## Figures and Tables

**Table 1:** Participants’ demographic data.

		*n*	%
Region	Turkish Republic of Northern Cyprus	8	4
	Turkey	192	96
Gender	Male	28	14
	Female	172	86
Age	20–30	94	47
	31–40	80	40
	41–50	23	11.5
	51–60	2	1
	≥60	1	0.5
Years of Practice In Pediatric Dentistry	0–5	92	46
	6–10	56	28
	11–20	46	23
	21–30	5	2.5
	≥31	1	0.5
Education Status	Pediatric Dentistry Specialist	120	60
	Postgraduate student in Pediatric Dentistry	80	40
Institution	State University Hospital	97	48.5
	State Clinic/Center/Hospital	16	8
	Private University Hospital	31	15.5
	Private Practice/Clinic/Hospital	56	28

**Table 2:** Participants’ working experience during the COVID-19 pandemic period.

Questions	Yes (*n*/%)	No (*n*/%)
Did you continue to live with your family while working during the COVID-19 lockdown period?	150 (75)	50 (25)
Have you had COVID-19 infection?	0	200 (100)
Have any of your family members had a COVID-19 infection?	3 (1.5)	197 (98.5)
Have any dentists and healthcare personnel working in your institution had a COVID-19 infection?	46 (23)	154 (77)
Have you attended a webinar on COVID-19?	110 (55)	90 (45)
Have you worked in the filiation team?	4 (2)	196 (98)
Did your working time decrease during this period?	192 (96)	8 (4)

**Table 3:** Knowledge of participants related to symptoms and transmission routes of COVID-19.

		*n* (%)
Symptoms	Fever	199 (99.5)
	Dry cough	194 (97.0)
	Shortness of breath	196 (98.0)
	Diarrhea	133 (66.5)
	Vomiting	93 (46.5)
	Runny nose	75 (37.5)
	Sore throat	158 (79.0)
	Sore eyes	102 (51.0)
	Skin Rash	70 (35.0)
	Joint or muscle pain	165 (82.5)
	Without Symptoms	181 (90.5)
	All of them	59 (29.5)
Transmission Routes	Direct transmission through: Coughing, sneezing	200 (100)
	Saliva	188 (94.0)
	Eye	170 (85)
	Blood	107 (53.5)
	Fecal–oral transmission	116 (58)
	Airborne transmission	175 (87.5)
	Indirect transmission through contact with fomites	179 (89.5)

**Table 4:** Knowledge of participants related to the statements about COVID-19.

Statement	Answer		
	True	False	I Don’t Know
Dental Healthcare workers are at high risk of being infected with COVID-19 when compared with the general population.	200 (100.0)	0 (0.0)	0 (0.0)
A single negative PCR test result does not exclude the possibility of COVID-19 infection among suspected patients.	195 (97.5)	3 (1.5)	2 (1.0)
Aerosol and droplets formed during dental treatment increase the risk of spread and transmission of COVID-19.	200 (100.0)	0 (0.0)	0 (0.0)
Pediatric patients present additional risks of transmission due to the use of appliances, difficulty in using personal protective equipment, and coming to the clinic with one or more parents.	197 (98.5)	3 (1.5)	0 (0.0)
Children can be asymptomatic or present mild, non-specific symptoms.	191 (95.5)	4 (2.0)	5 (2.5)
Asymptomatic patients and pre-symptomatic patients in the incubation period could be carriers of COVID-19.	198 (99.0)	0 (0.0)	2 (1.0)
All child patients and parents should be considered as potential carriers of COVID-19 unless proved otherwise.	194 (97.0)	5 (2.5)	1 (0.5)
Virus detection from saliva samples can be a diagnostic method.	150 (75.0)	11 (5.5)	39 (19.5)
Protection with mask is not recommended for children under 2 years of age and children unable to remove mask without assistance	115 (57.5)	27 (13.5)	58 (29.0)

**Table 5:** Infection control practices used in institutions of participants before dental treatment.

	*n*	(%)
Posting visual public notices for all visitors to the building entrances including signs and symptoms of COVID-19 and warning not to enter into the facility if they are exhibiting any of these symptoms.	156	78.8
Triaging dental patients by phone or online conferencing in order to decide the urgency of dental condition and COVID-19 risk status of patients.	120	60
Establishing pre-control staff at the institution for screening the temperature with a non-contact thermometer and checking appropriate use of face masks.	171	86.4
Questioning the travel history and presence of symptoms of everyone before entering the building.	143	72.2
Taking medical and dental anamnesis from pediatric patients and parents.	141	70.5
Placing hand sanitizer and asking children and parents to use it while entering the clinic.	145	73.2
Posting signs and posters at the entrance of the waiting room and in the areas visible to patients to provide instructions about social distancing, hand hygiene and respiratory hygiene measures.	162	81.8
Scheduling appointments of patients at times not close to each other in order to prevent crowding and establishment of the time required for disinfection and ventilation.	142	71.7
In the waiting room, applying social distance rules, and asking some of the patients to wait outside the building if necessary.	161	81.3
Ensuring that the pediatric patient comes to the clinic with a single accompanying person.	135	67.5
Removing toys or reading materials that could be touched by other children.	112	56.6

**Table 6:** Attitudes of participants when faced with a child patient or parent who had signs and symptoms of acute respiratory infection.

		What Would Your Attitude Be in Such a Situation?				*p*
		I Refer Them to the Hospital after Treating the Patient (*n*/%)	I Refer the Patient to the Hospital with Medical Mask without Conducting Treatment (*n*/%)	I Refuse to Treat the Patient and Ask Them to Leave the Clinic (*n*/%)	Total (*n*/%)	
Have you encountered a child patient or parent who had signs and symptoms of acute respiratory infection?	Yes	14 (31.8)	28 (63.6)	2 (4.5)	44 (100)	0.021
	No	24 (15.4)	110 (70.5)	22 (14.1)	156 (100)	

Pearson Chi-square test.

**Table 7:** Emergency situations encountered by participants during the COVID-19 pandemic period.

		*n*	%
Dental procedures performed during the COVID-19 lockdown period	Emergency dental treatments only	164	82
	Routine dental practices	6	3
	Both of them	37	18.5
Emergency dental treatment performed during the COVID-19 lockdown period	Severe pain caused by pulpal inflammation	188	94
	Pericoronitis, pain in the third molar region	0	0
	Abscess or bacterial infection causing localized pain and extraoral swelling	173	86.5
	Dental fractures causing pain or soft tissue injuries caused by trauma	71	35.5
	Luxations, dental avulsions	82	41
	Acute and painful lesions/ulcerations of the oral mucosa	39	19.5
	Dental treatments of oncology patients who are scheduled for organ transplantation	21	10.5
	Intraoral/extraoral infections that may compromise the patient’s airway	19	9.5
	Dental treatments required before general medical procedures	18	8
	Aerosol-free treatment of temporary restoration loss/fractures	54	27
	Maxillofacial trauma	17	8.5
	Adjustment of the orthodontic apparatus if it has caused ulceration or pain on the oral mucosa	41	20.5
	Life-threatening or uncontrolled oral tissue bleeding	6	3
	Suture removal	6	3
	Breakage of space maintainer	1	0.5

**Table 8:** Participants’ attitudes towards personal protective equipment (PPE) usage.

	Only in Aerosol-Generating Procedures		Used in Both Aerosol Generating and Non-Aerosol Generating Procedures		Never Used	
	*n*	%	*n*	%	*n*	%
Scrubs	11	5.5	174	87	15	7.5
Surgical mask	14	7	180	90	6	3.0
N95 respirator	42	21	102	51	56	28
P100 respirator	10	5	19	9.5	171	85.5
FFP2 respirator	32	16	87	43.5	81	40.5
FFP3 respirator	18	9	54	27	128	64
Goggles	26	13	142	71	32	16
Face shield	29	14.5	166	83	5	2.5
Elastomeric half mask	9	4.5	33	16.5	158	79
Disposable surgical gown	25	12.5	141	70.5	34	17
Disposable protective coverall	35	17.5	48	24	7	58.5
Disposable gloves	14	7	185	92.5	1	0.5
Disposable medical cap	23	11.5	167	83.5	10	5
Waterproof shoe cover	13	6.5	64	32	123	61.5

**Table 9:** Participants’ attitudes related to PPE.

		*n*	%
How did you explain your personal protective equipment to children?	I wore an astronaut suit	110	55
	I became a superhero	52	26
	I explained the reason	119	59.5
	Did not give any explanation	43	21.5
	Other	4	2
Do you think that personal protective equipment increases the anxiety levels of children?	Yes	174	87.0
	No	26	13.0
Have you paid attention to proper order for donning and doffing your personal protective equipment?	Yes	168	84.0
	No	32	16.0
Have you paid attention to removing your and other dental healthcare workers’ protective equipment in a separate isolation room?	Yes	110	55.0
	No	90	45.0
Have you ever encountered a situation such as burning, stinging, itching, dryness on your skin due to frequent hand cleaning and long-term use of personal protective equipment?	Yes	161	80.5
	No	39	19.5

**Table 10:** Infection control practices used by the participants during treatment.

		*n*	%
Which measures did you use while treating pediatric patients during the COVID-19 lockdown?	Not letting the parent into the treatment room	78	39.0
	Treatment of only one patient in a room	113	56.5
	Aerosol box	58	29.0
	Rubber dam	12	6.0
	High-speed handpiece and ultrasonic instruments	38	19.0
	Manual instruments, hand instrumentation	125	62.5
	Slow-speed handpieces	49	24.5
	Dental handpiece with anti-retraction function	5	2.5
	Chemomechanical caries removal	9	4.5
	Interim therapeutic restorations	71	35.5
	Four-handed technique	59	29.5
	Air water syringe	47	23.5
	Preprocedural oxidative or antimicrobial mouth-rinse	67	33.5
	High-volume saliva ejectors	69	34.5
	Dental extraoral suction system	13	6.5
	Providing two before three after hand hygiene	9	4.5
	Continuing patients’ control appointments via phone, WhatsApp and social networks	119	59.5
Have you obtained X-rays from your pediatric patients during the COVID-19 lockdown?	Yes, both intraoral and panoramic radiographs	48	24
	Yes, intraoral radiographs only	8	4
	Yes, only panoramic radiographs	119	59.5
	No	25	12.5
During the COVID-19 lockdown, did you have a pediatric patient who was not cooperative with non-pharmacological behavior management techniques?	Yes	129	64.5
	No	71	35.5

**Table 11:** Participants’ practice measures used with children who were not cooperative with non-pharmacological behavior management techniques during the COVID-19 pandemic period.

	*n*	(%)
Use of antibiotics and analgesics	145	72.5
Inhalation sedation	9	4.5
Enteral sedation	2	1.0
Parenteral sedation	2	1.0
General anesthesia	23	11.5
Physical restraining	16	8.0
Atraumatic restorative treatment	66	33.0
Hall technique	9	4.5
Chemomechanical caries removal	7	3.5
Laser applications	1	0.5
Silver diamine fluoride application	6	3.0
None of them	28	14.0

**Table 12:** Infection control practices used by the participants/institutions after treatment.

	*n*	(%)
Asking children and parents to use hand sanitizer when leaving the clinic.	104	52.5
Disinfecting reusable personal protective equipment with 70% alcohol after each usage.	136	68
Disinfection of commonly used areas such as dental unit, dental light, dental X-ray machine after each patient with 70% ethanol, 0.1% sodium hypochlorite, or 0.5% hydrogen peroxide.	133	66.5
Cleaning and sterilization of the dental hand instruments immediately after usage.	117	58.5
Ventilation of the treatment room after each patient.	170	85.0
Regular disinfection of common areas, door handles, chairs and tables with 0.1% sodium hypochlorite.	145	72.5
Discharge of medical waste in accordance with the legislation.	129	64.5
Fogging system for disinfection.	71	35.5
High-efficacy particulate air (HEPA) filtration system.	14	7.0
Ventilation and air-purifying System.	32	16
Ultraviolet radiation system.	18	9

## Data Availability

The data that support the findings of this study are available on request from the corresponding author.

## Supplementary Materials

The following are available online at https://www.mdpi.com/article/10.3390/medicina57111140/s1. Supplementary File S1: Survey Questionnaire.
