# Supplementary material for: Clinical Experience, Knowledge, Attitudes and Practice of Turkish Pediatric Dentists during the COVID-19 Pandemic
Source: Medicina (Kaunas). 2021 Oct 21;57(11):1140. doi: 10.3390/medicina57111140 (PMC8618506; doi:10.3390/medicina57111140)
Supplement: Supplementary file 1 [file medicina-57-01140-s001.zip › medicina-1392540-supplementary.pdf]

Supplementary File S1: Survey Questionnaire

Part 1

1. Where are you working?
   - Turkey
   - Turkish Republic of Northern Cyprus

2. What is your age?
   - 20-30
   - 31-40
   - 41-50
   - 51-60
   - 60+

3. What is your gender?
   - Female
   - Male

4. How many years have you been practicing in the field of pediatric dentistry?
   - 0-5
   - 6-10
   - 11-20
   - 21-30
   - 31+

5. Please select your education level

- Postgraduate student in Paediatric Dentistry.
- Paediatric Dentistry Specialist

6. Please select your institution

- State University Hospital
- State Clinic / Centre Hospital
- Private University Hospital
- Private Practice / Clinic / Hospital

7. Did you continue to live with your family while working during the COVID-19 lockdown period?.

- Yes
- No

8. Have you had COVID-19 infection?

- Yes
- No

9. Have any of your family members had a COVID-19 infection?

- Yes
- No

10. Have any dentists and healthcare personnel working in your institution had a COVID-19 infection?

- Yes
- No

11. Have you attended a webinar program on COVID-19?

- Yes
- No

12. Have you worked in the Filiation team?

- ◯ Yes
- ◯ No

13. Did your working time decrease during this period?

- ◯ Yes
- ◯ No

Part 2:

14. What are the symptoms of COVID-19 infection?

- ☐Fever
- ☐Dry Cough
- ☐Shortness of breath
- ☐Diarrhoea
- ☐Vomiting
- ☐Runny nose
- ☐Sore throat
- ☐Sore eyes
- ☐Skin Rash
- ☐Joint or muscle pain
- ☐Without Symptoms
- ☐All of them

15. What are the routes of transmission of COVID-19 infection?

☐Direct Transmission through: Coughing, sneezing

☐Saliva

☐Eye

☐Blood

☐Faecal-oral Transmission

☐Airborne Transmission

☐Indirect Transmission through contact with fomites

16. Dental Healthcare workers are at high risk of being infected with COVID-19 when compared with the general population.

- True
- False
- I don’t know

17. A single negative PCR test result does not exclude the possibility of COVID-19 infection among suspected patients.

- True
- False
- I don’t know

18. Aerosol and droplets formed during dental treatment increase the risk of spread and transmission of COVID-19

- True
- False
- I don’t know

19. Paediatric patients present additional risks of transmission due to the use of appliances, difficulty in using personal protective equipment, and coming to the clinic with one or more parents.

- True
- False
- I don’t know

20. Children can be asymptomatic or present mild, non-specific symptoms

- True
- False
- I don’t know

21. Asymptomatic patients and pre-symptomatic patients in the incubation period could be carriers of COVID-19.

- True
- False
- I don't know

22. All child patients and parents should be considered as potential carriers of COVID- 19 unless proved otherwise.

- True
- False
- I don't know

23. Virus detection from saliva samples can be a diagnostic method.

- True
- False
- I don't know

24. Protection with mask is not recommended for children under 2 years of age and children unable to remove mask without assistance.

- True
- False
- I don't know

Part 3:

25. Have you encountered a child patient or parent who had signs and symptoms of acute respiratory infection?

- Yes
- No

26. What would your attitude be in such a situation?

☐I refer them to the hospital after treating the patient
☐I refer the patient to the hospital with medical mask without conducting treatment
☐I refuse to treat the patient and ask them to leave the clinic.

27. Which personal protective equipment (PPE) did you use during the COVID-19 Pandemic period?

	Only in aerosol Generating Procedures	Used in both aerosol generating and non-aerosol generating procedures	Never used
Scrubs	⬭	⬭	⬭
Surgical mask	⬭	⬭	⬭
N95 respirator	⬭	⬭	⬭
FFP2 respirator	⬭	⬭	⬭
FFP3 respirator	⬭	⬭	⬭
P100 respirator	⬭	⬭	⬭
Goggles	⬭	⬭	⬭
Face shield	⬭	⬭	⬭
Elastomeric half mask	⬭	⬭	⬭
Disposable suurgical gown	⬭	⬭	⬭
Disposable protective coverall	⬭	⬭	⬭
Disposable gloves	⬭	⬭	⬭
Disposable medical cap	⬭	⬭	⬭
Disposable medical cap	⬭	⬭	⬭
Waterproof shoe cover	⬭	⬭	⬭

28. How did you explain your personal protective equipment to children?

- ☐ I wore an astronaut suit
- ☐I became a super hero
- ☐I explained the reason
- ☐Did not give any explanation
- ☐Other

29. Do you think that personal protective equipment increases the anxiety levels of children?

- Yes
- No

30. Have you paid attention to proper order for donning and doffing your personal protective equipment?

- Yes
- No

31. Have you paid attention to removing your and other dental health care workers' protective equipment in a separate isolation room?

- Yes
- No

32. Have you ever encountered a situation such as burning, stinging, itching, dryness on your skin due to frequent hand cleaning and long-term use of personal protective equipment?

- Yes
- No

Part 4:

33. Which dental procedures did you performed during the COVID-19 lockdown period?

☐Emergency dental treatments only

☐Routine Dental Practices

☐Both of them

34. Which emergency dental treatments did you performed during the COVID-19 lockdown period?

☐Severe pain caused by pulpal inflammation

☐Pericoronitis, pain in the third molar region

☐Abscess or bacterial infection causing localized pain and extraoral swelling

☐Dental fractures causing pain or soft tissue injuries caused by trauma

☐Luxations, dental avulsions

☐Acute and painful lesions / ulcerations of the oral mucosa

☐Dental treatments of oncology patients and who are scheduled for organ transplantation

☐Intraoral/extraoral infections that may compromise the patient's airway

☐Dental treatments required before general medical procedures

☐Aerosol-free treatment of temporary restoration loss / fractures

☐Maxillofacial trauma

☐Adjustment of the orthodontic apparatus if it has caused ulceration or pain on the oral mucosa

☐Life-threatening or uncontrolled oral tissue bleeding

☐Suture removal
☐Breakage of space maintainer

Part 5:

35. Which infection control measures used in your institution before dental treatments.during the COVID-19 lockdown?

☐Posting visual public notices for all visitors to the building entrances including signs and symptoms of COVID-19 and warning not to enter into the facility if they are exhibiting any of these symptoms.
☐Triaging dental patients by phone or online conferencing in order to decide the urgency of dental condition and COVID-19 risk status of patients.
☐Establishing pre-control staff at the institution for screening the temperature with a non-contact thermometer and checking appropriate use of face masks.
☐Questioning the travel history and presence of symptoms of everyone before entering the building
☐Taking medical and dental anamnesis from paediatric patients and parents
☐Placing hand sanitizer and asking children and parents to use it while entering the clinic
☐Posting signs and posters at the entrance of the waiting room and in the areas visible to patients to provide instructions about social distancing, hand hygiene and respiratory hygiene measures.
☐Scheduling appointments of patients at times not close to each other in order to prevent crowding and establishment of the time required for disinfection and ventilation.
☐In the waiting room, applying social distance rules, and asking some of the patients to wait outside the building if necessary
☐Ensuring that the paediatric patient comes to the clinic with a single accompanying person.
☐Removing toys or reading materials that could be touched by other children

36. Which measures did you use while treating paediatric patients during the COVID-19 lockdown?

☐Not letting the parent into the treatment room
☐Treatment of only one patient in a room
☐Aerosol box
☐Rubber dam
☐High speed handpiece and ultrasonic instruments
☐Manual instruments, hand instrumentation
☐Slow speed handpieces
☐Dental handpiece with anti-retraction function
☐Chemomechanical caries removal
☐Interim Therapeutic Restorations
☐4 handed technique
☐Air Water Syringe
☐Preprocedural oxidative or antimicrobial mouth-rinse
☐High-volume saliva ejectors
☐Dental extra oral suction system
☐Providing two before three after hand hygiene
☐Continuing patients' control appointments via phone, WhatsApp and social networks

37. Have you obtained X-rays from your paediatric patients during the COVID-19 lockdown?

☐Yes, both intraoral and panoramic radiographs

☐Yes, intraoral radiographs only

☐Yes, only panoramic radiographs

☐No

38. During the COVID-19 lockdown, did you have a paediatric patient who was not cooperative with non-pharmacological behaviour management techniques?

◯ Yes

◯ No

39. Which measures did you use for dental treatments of children who were not cooperative with non-pharmacological behaviour management techniques during the COVID-19 pandemic period.

☐Inhalation sedation

☐Enteral sedation

☐Parenteral sedation

☐General anaesthesia

☐Physical restraining

☐Atraumatic restorative treatment

☐Hall technique

☐Chemomechanical caries removal

☐Laser applications

☐Silver diamine fluoride application

☐None of them

40. Which infection control measures used in your institution after dental treatments.during the COVID-19 lockdown?

☐Asking children and parents to use hand sanitizer when leaving the clinic

☐Disinfecting reusable personal protective equipment with 70% alcohol after each usage

☐Disinfection of commonly used areas such as dental unit, dental light, dental X-ray machine after each patient with 70% ethanol, 0.1% sodium hypochlorite, or 0.5% hydrogen peroxide

☐Cleaning & sterilization of the dental hand instruments immediately after usage

☐Ventilation of the treatment room after each patient

☐Regular disinfection of common areas, door handles, chairs and tables with 0.1% sodium hypochlorite

☐Discharge of medical waste in accordance with the legislation

☐Fogging system for disinfection

☐High Efficacy Particulate Air (HEPA) Filtration System

☐Ventilation and air-purifying system

☐Ultraviolet radiation System
